# Silk Fibroin Hydrogel Reinforced With Magnetic Nanoparticles as an Intelligent Drug Delivery System for Sustained Drug Release

**DOI:** 10.3389/fbioe.2022.891166

**Published:** 2022-07-15

**Authors:** Mahsa Haghighattalab, Abdolmohammad Kajbafzadeh, Mostafa Baghani, Ziba Gharehnazifam, Bahareh Mohammadi Jobani, Majid Baniassadi

**Affiliations:** ^1^ School of Mechanical Engineering, College of Engineering, University of Tehran, Tehran, Iran; ^2^ Department of Urology, School of Medicine, Tehran University of Medical Sciences, Tehran, Iran; ^3^ Pediatric Urology Research Center, Children’s Medical Center, Tehran, Iran

**Keywords:** silk fibroin, magnetic hydrogel, intelligent drug delivery, remote stimulation, sustained drug release

## Abstract

Due to the well-known biocompatibility, tunable biodegradability, and mechanical properties, silk fibroin hydrogel is an exciting material for localized drug delivery systems to decrease the therapy cost, decrease the negative side effects, and increase the efficiency of chemotherapy. However, the lack of remote stimuli response and active drug release behavior has yet to be analyzed comparatively. In this study, we developed magnetic silk fibroin (SF) hydrogel samples through the facile blending method, loaded with doxorubicin hydrochloride (DOX) and incorporated with different concentrations of iron oxide nanoparticles (IONPs), to investigate the presumable ability of controlled and sustained drug release under the various external magnetic field (EMF). The morphology and rheological properties of SF hydrogel and magnetic SF hydrogel were compared through FESEM images and rheometer analysis. Here, we demonstrated that adding magnetic nanoparticles (MNPs) into SFH decreased the complex viscosity and provided a denser porosity with a bigger pore size matrix structure, which allowed the drug to be released faster in the absence of an EMF. Release kinetic studies show that magnetic SF hydrogel could achieve controlled release of DOX in the presence of an EMF. Furthermore, the drug release from magnetic SF hydrogel decreased in the presence of a static magnetic field (SMF) and an alternating magnetic field (AMF), and the release rate decreased even more with the higher MNPs concentration and magnetic field strength. Subsequently, Wilms’ tumor and human fibroblast cells were cultured with almost the same concentration of DOX released in different periods, and cell viability was investigated using MTT assay. MTT results indicated that the Wilms’ tumor cells were more resistant to DOX than the human fibroblasts, and the IC50 values were calculated at 1.82 ± 0.001 and 2.73 ± 0.004 (μg/ml) for human fibroblasts and Wilms’ tumor cells, respectively. Wilms’ tumor cells showed drug resistance in a higher DOX concentration, indicating the importance of controlled drug delivery. These findings suggest that the developed magnetic SFH loaded with DOX holds excellent potential for intelligent drug delivery systems with noninvasive injection and remotely controlled abilities.

## GRAPHICAL ABSTRACT

Preparation and magnetic stimulation of SFH-DOX/MNPs for in vitro drug release study.
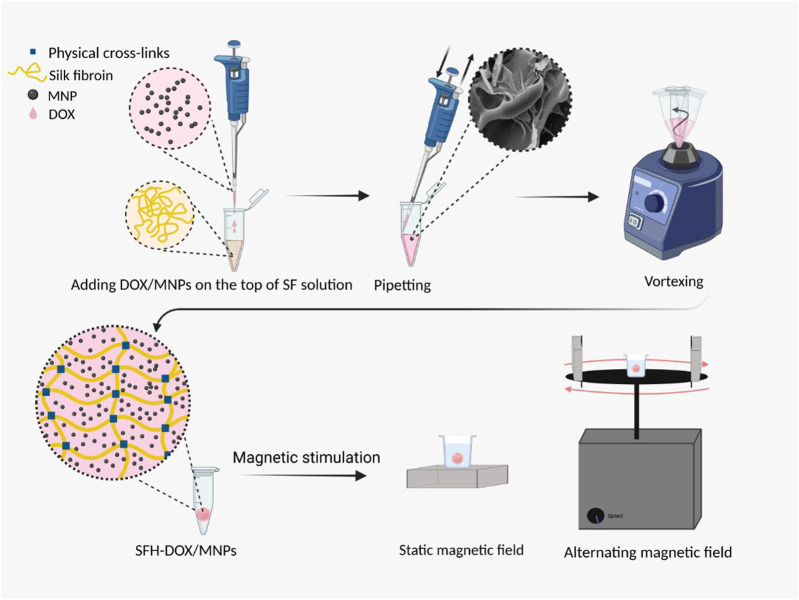

## 1 Introduction

Cancer is one of the leading causes of morbidity and mortality worldwide (Fitzmaurice et al., 2015), and Wilms’ tumor is the most common kidney cancer in young children, diagnosing 500 to 600 new cases each year in the united states alone (American Cancer Society, 2020). The main cancer treatments involve surgery, chemotherapy, and radiotherapy (Zitvogel et al., 2008; Bhatnagar, 2009). Despite many drugs and treatments developed, one of the problems in chemotherapy is the side effects of systemic chemotherapy, which is caused by free drug distribution, nonspecific targeting, short blood circulation half-life, fast *in vivo* metabolization, and drug resistance (Tian et al., 2014). By now, many nanometer-scale drug delivery systems based on natural and synthetic polymers have been developed to ameliorate these issues (Sung and Kim, 2020; Yao et al., 2020). Interestingly, silk is a natural, abundant, and reasonable cost polymeric source of biomaterials, demonstrating extraordinary mechanical properties, biocompatibility, and tunable biodegradability (Nguyen et al., 2019; Tomeh et al., 2019; Kochhar et al., 2021). Silk is composed of two main proteins, sericin, and fibroin. Despite having many biological activities, such as anti-bacterium, anti-coagulation, anti-oxidation, and promoting cell growth and differentiation (Wang et al., 2014), sericin is commonly extracted because of its combination with fibroin invokes an inflammatory response (Aramwit et al., 2009; Lam et al., 2021). Silk fibroin (SF) materials have been used as a desirable biomaterial, due to their physicochemical and mechanical properties, for several applications in biomedical and pharmaceutical through the past decades, such as small molecule drug delivery (Seib et al., 2013; Zhong et al., 2015; Gharehnazifam et al., 2021), gene delivery (Huang et al., 2016), biological drug delivery (Guziewicz et al., 2011), wound healing (Chouhan et al., 2018; He et al., 2019; Maaz Arif et al., 2021; Vidya and Rajagopal, 2021), cartilage regeneration (Cheng et al., 2018), and bone generation (Fini et al., 2005; Jin et al., 2015; Yan et al., 2019). SF as a biomaterial can be implemented in various forms of fabrication such as sponges, tubes, fibers, films, microspheres, and hydrogels as implantable and injectable systems (Rockwood et al., 2011). SF hydrogels have a shear-thinning behavior (Guziewicz et al., 2011) that provides the ability of injection and can be used as a career for a drug to provide bioavailability and sustainable release in the tumor site. Responding to the need to lower chemotherapy’s side effects, many researchers have utilized localized chemotherapy with a controlled and sustained drug release, using regulating endogenous parameters (Pritchard and Kaplan, 2011; de Moraes et al., 2015; Harris et al., 2016; Zuluaga-Vélez et al., 2019) or with SF’s different micro-environmental stimuli response properties hydrogel (Gou et al., 2020). However, controlling the release profile through remote stimuli is still an issue, leading to greater control to optimize the therapy. One strategy for remote controlling the drug release is using ferrogels, which can be prepared by introducing magnetic nanoparticles (MNPs) to the polymeric chain of the hydrogel to create a magneto-responsive hydrogel (Frachini and Petri, 2019). The previous researchers investigated the drug release profile of many different ferrogels (Liu et al., 2006; Hu et al., 2018). However, there have been no studies on magnetic silk-based hydrogel drug release behavior to the best of our knowledge.

This study aims to investigate the ability of sustained and remotely controlled release of the drug encapsulated in the magnetic SF hydrogel, using the regulation of external magnetic field (EMF) and MNPs concentration. For this purpose, Iron oxide nanoparticles (IONPs) were introduced to the SF solution through the blending method (Frachini and Petri, 2019), and the drug model was encapsulated in the composition using physical crosslinks. Physical crosslinking is safe, inexpensive, and has less toxicity for cells than crosslinking using chemical agents; thus, it is particularly of interest (Johari et al., 2020).

IONPs have different biomedical applications, such as diagnosis and treatment, and have been widely used as contrast agents for bioimaging, magnetic hyperthermia agents, and drug delivery (Xu C. et al., 2019). Moreover, IONPs have superparamagnetic properties (less than 50 nm) (Frachini and Petri, 2019) and are clinically approved (10–300 nm) (Jin et al., 2014). Doxorubicin hydrochloride (DOX) has been taken as a drug model, mainly used to treat Wilms’ tumor (Green et al., 2007; Malogolowkin et al., 2008; Spreafico et al., 2009; Oostveen and Pritchard-Jones, 2019), which usually causes issues like drug resistance and free drug distribution (Smith et al., 2006; Christowitz et al., 2019). Samples of SF hydrogel loaded with DOX and MNPs were developed to address these problems and were used as drug delivery systems to monitor the drug release rate profile. This approach makes magnetic SF hydrogel a multi-responsive system for sustained and controlled drug release.

**FIGURE 1:**
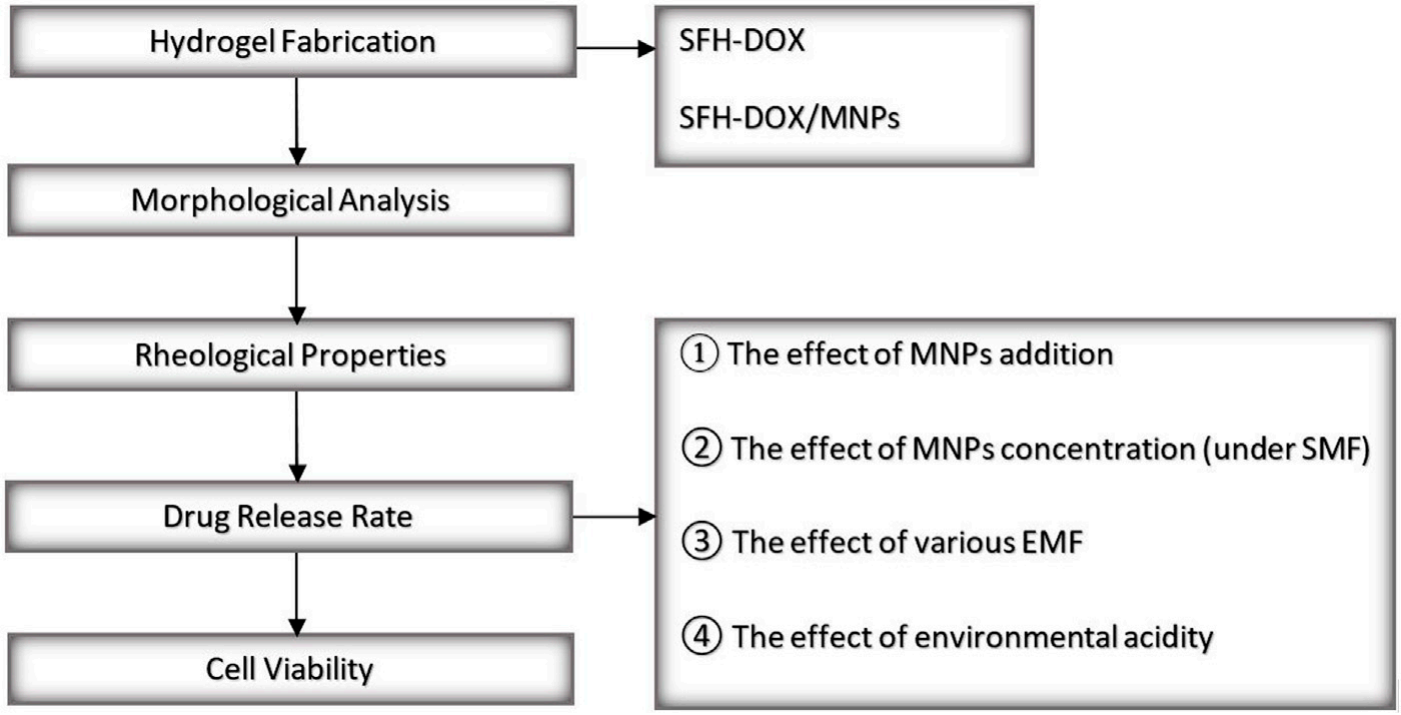
An overview of the present study.

A field emission scanning electron microscope (FESEM) was conducted to investigate the morphology of SF solution in comparison with SF/MNPs solution, which has a critical role in the release behavior of drugs from the SF scaffolds. In general, the morphology of hydrogels can reflect vital characteristics such as swelling behavior and viscosity (Park et al., 2013; Hu et al., 2017; Hu et al., 2018; Tanasić et al., 2021). Moreover, a rheometer was used for the study of the rheological properties of SF hydrogel and the effect of the addition of MNPs on its viscoelasticity and mechanical strength, which is mainly related to the crosslink density (Piluso et al., 2020). The rate of the drug released was monitored during different periods using spectrophotometry. Shortly, the effect of different parameters on the drug release rate was evaluated through different scenarios. Investigated parameters include; 1) the addition of MNPs to the SF hydrogel in passive mode, 2) applying different EMFs on the magnetic SF hydrogel, 3) the concentration of MNPs while applying an EMF, and 4) environmental pH. In the end, the bioactivity of the released DOX was accessed *in vitro* on human fibroblast and Wilms’ tumor cells through an MTT assay to evaluate and compare the cell death in the exposure of different Dox concentrations released in different time intervals from scaffolds. An overview of the present study is illustrated in Figure 1.

## 2 Materials and Methods

### 2.1 Materials

Bombyx Mori cocoons were supplied by a local market (from Chamkhaleh, Gilan, Iran). Collagenase type II, dispase, fetal bovine serum (FBS), Dulbecco’s Modified Eagle Medium and ham’s F12 medium (DMED-F12), Penicillin/streptomycin (10.000 U/ml) , and Trypsin-EDTA 1X were purchased from Gibco, U.S.A. Dimethyl sulfoxide (DMSO) and thiazolyl blue tetrazolium bromide (MTT powder) were produced from Sigma – -Aldrich, Germany. Lithium bromide (LiBr) was supplied from Merk, Germany. Bovine Serum Albumin was purchased from Biowest, Nuaille, France. Phosphate buffered saline (PBS) 1X, and sodium carbonate (Na2CO3) were purchased from DNAbiotech, Iran. Dispersable IONPs (Fe3O4 with a mean spherical size of 20–30 nm) were obtained from US Research, U.S.A. Deionized water was used throughout the experimental process.

### 2.2 Silk Fibroin Solution

The preparation of the SF solution was based on the previously described protocol by Kaplan and coworkers (Rockwood et al., 2011). Briefly, *Bombyx mori* cocoons were cut into small pieces after cleaning out of warm. Silk cocoons were boiled for 30 min in 0.02 M Na2CO3 solution to remove sericin protein. Then, degummed silk fibers were washed for 20 min three times with ultrapure water, squeezed, and allowed to air-dry overnight at room temperature. Parameters in the degumming process, such as time, Na2CO3 concentration, volume, and temperature, have an essential impact on resultant silk fibroin mechanical properties, molecular weight, and crystallinity, which can be optimized for different purposes (Seib et al., 2013; Bucciarelli et al., 2021). In the next step, SF fibers were dissolved in 9.3 M LiBr solution and were incubated at 62°C for 4 h, followed by dialyzing the solution against deionized water in a dialysis membrane [molecular weight cut-off (MWCO) 12,000 Da] for 48 h, following by centrifuging two times for 20 min in 4°C and 9,000 rpm. The resulting solution had an approximate concentration of 8% (Wt/Vol) and was kept at 4°C for 1 month for further use.

### 2.3 DOX-Loaded Magnetic Silk Fibroin Hydrogel

DOX-loaded magnetic SF hydrogel was prepared in the fast and facile blending method (Li et al., 2013; Frachini and Petri, 2019). 25, 50, and 75 (μg) of Fe3O4 magnetic nanoparticles (Tian et al., 2014) were added to 100 μL of DOX 50mg/25mL (Pfizer, Australia) and mechanically mixed. The homogenous mixture was added to the SF solution and mechanically mixed before vortexing or sonicating gelation. The gelation process is fast enough to prevent MNPs from sinking. SF solution also acts as a coating layer; thus, MNPs are hindered from undergoing agglomeration. DOX was added to the SF solution with a ratio of 1:3 in all samples (Seib et al., 2013). For the gelation process, samples were vortexed for 20 min for the *in vitro* release study and sonicated (5 s at 20% amplitude) in a mold for the rheological test until the gelation occurred.

### 2.4 Field Emission Scanning Electron Microscope Observation

#### 2.4.1 Silk Fibroin Sample

SF Solution was mixed with water in the ratio of 1:3 and freeze-dried using liquid N2 after being frozen. Water was added to the SF solution to liken the condition to the situation where SF hydrogels are loaded with a drug.

#### 2.4.2 Magnetic Silk Fibroin Sample

25 μ g of Fe3O4 powder were added to 100 μl water and homogeneously mixed with SF solution with the ratio of 1:3, being kept in a Petri dish, frozen, and freeze-dried with liquid N2.

#### 2.4.3 Morphological Analysis

The morphology of SF and magnetic SF samples was observed using a HITACHI S4160 field emission scanning electron microscope (FESEM) (Tokyo, Japan). The samples were freeze-dried under identical conditions, coated with a conductive adhesive layer, and sprayed with a layer of gold to electrically conductive, for higher image resolution, photographed with an accelerating voltage of 20 kV at room temperature.

### 2.5 Rheological Characterization

The rheological properties of SFH-DOX and SFH-DOX/MNP1 were measured for comparison using an MCR 502 rheometer (Anton Paar, Hertford Herts, United Kingdom) at a controlled temperature of 37°C. 3 ml of SF-DOX and SF-DOX/MNP1 solutions were sonicated gelled (5 s at 20% amplitude) in a cylindrical mold with a diameter of 25 mm and a height of 3 mm and incubated at 37°C until the gelation occurred eventually (12 h). For the frequency sweeping test, complex viscosity $\left(\eta^{*}\right)$ , storage moduli $\left(G^{\prime }\right)$ , and loss moduli $\left(G^{\prime \prime }\right)$ were monitored over a wide angular frequency range (ω=0.1−1000 rad/s) at a fixed constant strain (γ= 0.2%) (Hasturk et al., 2020). The examinations were operated in parallel-plate configuration, with a diameter of 25 mm and at a 1.5 mm gap distance.

### 2.6 *In Vitro* Drug Release Study

To determine the release profile of DOX, 200 μL of each sample vortexed for 20 min and incubated at 37°C for 12 h to completely gel. Then, samples were soaked in 1.5 ml of PBS 10X into a 24-well cell culture plate and then incubated at 37°C, allowing the release of the drug. At a specific time interval (up to 35 days) (Seib et al., 2013), the solvent was periodically collected for analysis and replaced with the same amount of fresh PBS. DOX concentrations were quantified by UV-vis spectrophotometer—NanoDrop 2000 (Thermofisher Scientific, US) at $\lambda_{max}$ 500 nm. A standard curve (absorbance-concentration) was drawn using the detection of absorbance of ten samples with known concentrations. With fitting a linear curve (R^2 = 0.98250865, St. error for Xn = 5.32448 e^-05), all unknown concentrations were calculated. All studies were done in triplicates. The DOX release percentage (Equation 1) and cumulative drug release were investigated as a function of incubation time.

$$\text{DOX}\;\text{release}\;\text{percentage}=\;\frac{\text{DOX}\;\text{amount}\;\text{in}\;\text{the}\;\text{supernatant }}{\text{The}\;\text{initial}\;\text{DOX}\;\text{amount}\;\text{in}\;\text{the}\;\text{hydrogel }}\;\times 100.$$ (1)

A total of eight different samples were used for evaluating the effect of the following parameters on the drug release rate.

1) Addition of MNPs: to independently evaluate the effects of the adding of MNPs on the passive DOX release, SFH-DOX, and SFH-DOX/MNP1 were incubated in the absence of any magnetic field in PBS 10X (pH 6.76) at 37°C.
2) EMF: to observe the effect of applying a magnetic field on the release rate, drug release from SFH-DOX/MNP1 in the absence of EMF is considered as control and compared with those of 0.18 T static magnetic field (SMF1), 0.28 T static magnetic field (SMF2), and 5 mT alternating magnetic field (AMF1) were applied. SMF1 and SMF2 were stationarily incubated with the hydrogel, while AMF1 was pulsed and applied for an hour (10 min on-5 min off) before each sample collection time point. The SMFs were created using neodymium magnets (30 cm length × 20 cm width × 10 cm thickness, and 50 cm length × 30 cm width × 10 cm thickness) and were placed under the 24-well plate. The magnetic field strength on the bottom of the sample well was measured at approximately 0.18 and 0.28 T for SMF1 and SMF2, respectively. The AMF was created by placing two neodymium magnets (40 cm length × 20 cm width × 10 cm thickness) on a rotary plate at a distance of 10 cm from each other. The setup was placed around the sample, the frequency was 2 kHz, and the magnetic field strength was measured, changing from 0 to 5 mT at the sample surface (the experimental setup of the AMF showed in the graphical abstract).
3) MNPs concentration: to observe the effect of MNPs concentration on drug release profile, SFH-DOX/MNP1, SFH-DOX/MNP2, and SFH-DOX/MNP3 were compared in the presence of SMF2. Accurate details of sample components and conditions used in different scenarios are mentioned in Table 1.
4) Environmental pH: the effect of medium pH on *in vitro* drug release rate was investigated through soaking SFH-DOX/MNP1 in PBS 10X, with two different pHs 6.76 and 7.49.

**TABLE 1:** Nomenclature, composites, and properties of applied external actuation of silk fibroin hydrogel.

Nomenclature	SF solution	DOX	MNPs	EMF
SFH-DOX	150 μL	50 μL	0	0
SFH-DOX/MNP1	150 μL	50 μL	12.5 μg	0
SFH-DOX/MNP1-AMF1	150 μL	50 μL	12.5 μg	AMF 0.05 T, frequency 2 Hz
SFH-DOX/MNP1-SMF1	150 μL	50 μL	12.5 μg	SMF 0.18 T
SFH-DOX/MNP1-SMF2	150 μL	50 μL	12.5 μg	SMF 0.28 T
SFH-DOX/MNP2-SMF2	150 μL	50 μL	25 μg	SMF 0.28 T
SFH-DOX/MNP3-SMF2	150 μL	50 μL	37.5 μg	SMF 0.28 T

### 2.7 Cell Culture

Human fibroblast cells (isolated from the foreskin by enzymatic digestion) and Wilms’ tumor cells (by explant culture) were cultured in DMEM-F12 containing 10% (FBS) and 1% penicillin and streptomycin. All cells were incubated in a humid incubator at 37°C with 5% CO_2_ and 95% humidity, and trypsin-EDTA passaged at 70–90% confluence. Human fibroblast and Wilms’ tumor cells were used up to the third and fourth passage, respectively.

### 2.8 *In vitro* Cytotoxicity Assay

MTT assay was employed to quantification of cell viability. This method is based on the reduction of the tetrazolium salt solution (MTT) to purple formazan by metabolically active cells. Human fibroblast and Wilms’ tumor cells were plated in 96-well plates at an initial density of $7\times 10^{3}$ cells per 200 μL cell culture medium and were allowed to adhere for 4 h at 37°C, 5% CO_2,_ and 95% humidified atmosphere. Human fibroblast cells were taken as a control for assessing and comparing the response of normal healthy cells and Wilms’ tumor cancerous cells with DOX exposure. Cells were exposed to seven different concentrations (0 as control, 0.91, 1.82, 2,73, 3.64, 5.45, 6.36 μL/mL ) of DOX for 48 h, with quadruplicate of each sample. DOX dose values were determined from the drug release profile and are approximately equaled to the concentration of the released drug in different periods.

In the next step, the culture medium was aspirated, 180 μL of medium (without FBS), and 20 μL of sterile MTT color (5  mg/mL in PBS) were added to each well, followed by 4 h of incubation. The culture medium was aspirated, and 150 μL DMSO was added to each well. After 10 min of shaking the plates in the darkness, the formed formazan crystal was solubilized in DMSO, and the optical density (OD) of each concentration was evaluated in the wavelength of 490 and 630 nm using an ELISA reader (Synergy HT, Biotech, United States). Cells without drug exposure were used as controls, and their absorbance values were subtracted from those corresponding to the contact with the drug. The percentage of viable cells was calculated from relation 2) (actual relative ratio), and half-maximal inhibitory concentration (IC50) was computed with SigmaPlot software version 12.

$$\text{\%}\;viable\;cells=\frac{\left(abs_{sample}-abs_{blank}\right)}{\left(abs_{control}-abs_{blank}\right)}\times 100.$$ (2)

## 3 Results and Discussion

### 3.1 Morphology Observation

**FIGURE 2:**
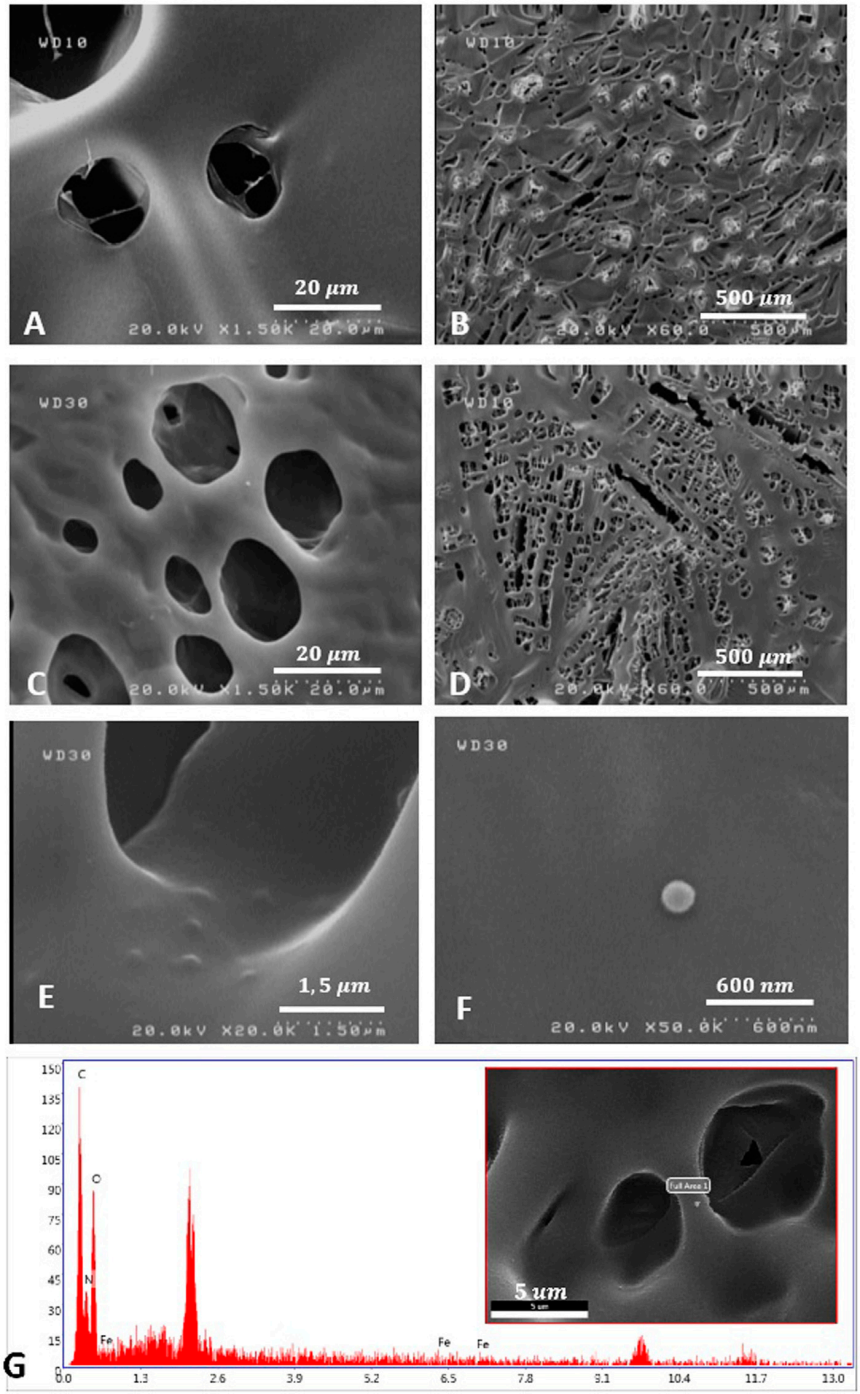
The morphology observation of SFH **(A**,**B)**, and SFH/MNP1 **(C–F)** using FESEM. **(G)** Material characterization of SFH/MNP1 using Energy Dispersive X-Ray Analysis (EDAX) in the selected area.

The freeze-dried SF solutions were prepared to observe the FESEM appearance. The morphology of the SF solution and SF/MNP1 solution is presented in Figure 2. The FESEM results exhibited that the internal structure of the SF and magnetic SF is a porous three-dimensional network structure with an irregular pore shape, and pore arrangement had no particular direction. FESEM images indicated that SF and magnetic SF have a porous fibrillary matrix, allowing the drug to release through the matrix. Furthermore, the average diameter of the pores and porosity seemed to increase with the addition of Fe3O4 nanoparticles in the SF solution. Quantitative measurements were assessed using ImageJ software. The porosity of SFH and SFH/MNP1 lyophilized solution was calculated at 14.306 ± 7.052% and 23.59 ± 8.123%, respectively. Data are expressed as mean ± SD for at least ten random areas. Regarding pore area, pore size increased from the average size of 154.764 $\mu m^{2}$ to 188.299 $\mu m^{2}$ , with the addition of magnetic nanoparticles. Moreover, the increase in pore size and porosity density can be explained by the difference in the hydrogel structure’s viscosity and swelling properties. The higher the viscosity, the more unfavorable the motion of water. Thus, smaller ice particles form while freeze-drying, and ice crystals cannot grow due to the resistance. Moreover, lower water uptake resulted in smaller ice crystals in the lyophilization process, which was eventually represented by smaller pores (Li et al., 2001; Hu et al., 2017; Hu et al., 2018). In addition, an increase in pore size could be explained by the decrease in intermolecular hydrophobic interaction with adding MNPs, resulting in more water intake (Park et al., 2013).

### 3.2 Rheological Properties

**FIGURE 3:**
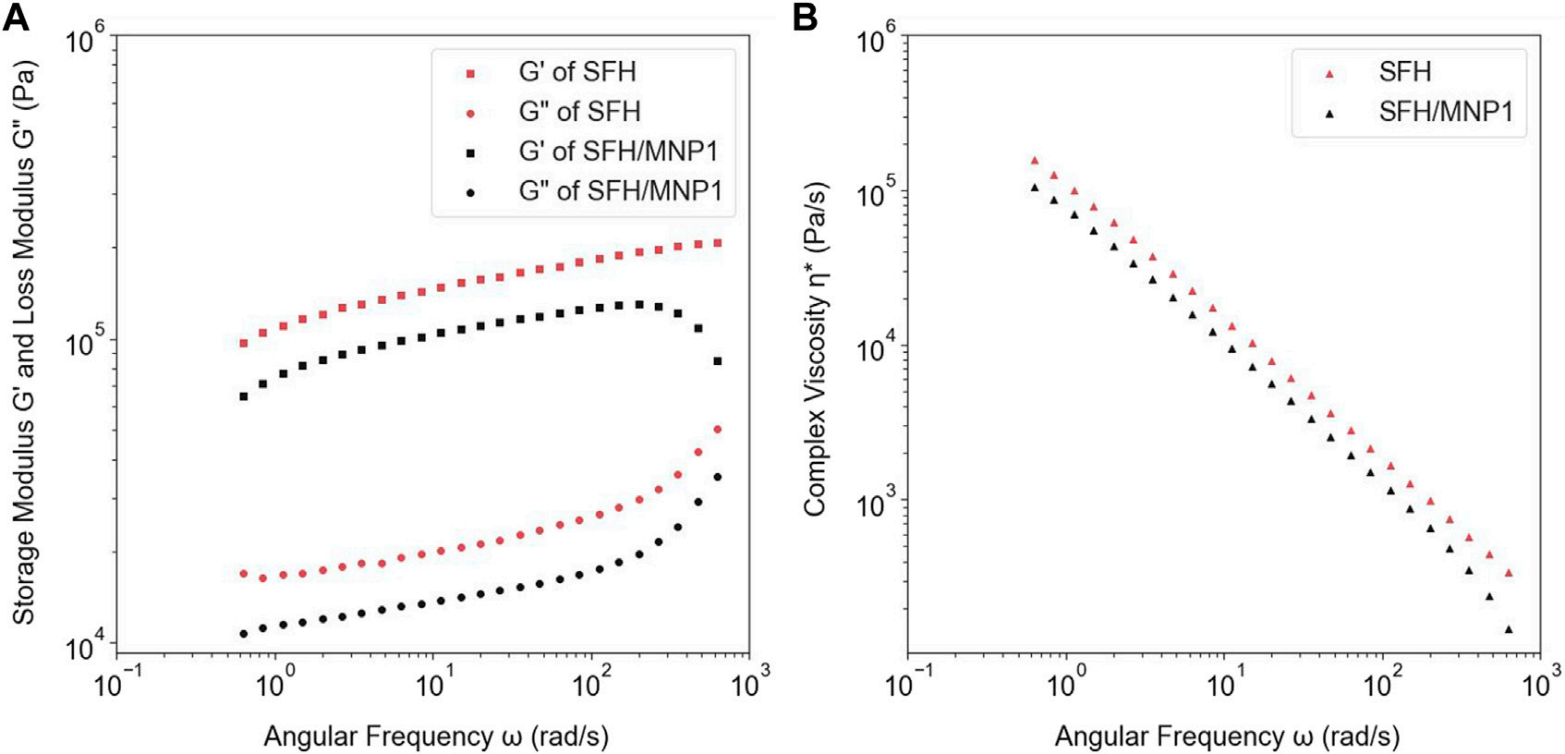
**(A)** The comparison between the storage and loss modulus of SFH-DOX and SFH-DOX/MNP1. **(B)** The changes of complex viscosity of SFH-DOX and SFH-DOX/MNP1 during the different angular frequencies.

An angular frequency sweep was performed to analyze the physical nature of hydrogels. The dynamic complex viscosity $\left(\eta^{*}\right)$ , storage moduli $\left(G^{\prime }\right)$ , and loss moduli $\left(G^{\prime }\right)$ , as a function of the angular frequency (ω) of SFH-DOX and SFH-DOX/MNP1 are reported in Figure 3. $\;G^{\prime }$ and $G^{\prime \prime }$ indicate energy stored and recovered, and energy loss per cycle, respectively. As viscoelastic materials have a combination of elasticity and viscosity behavior $\;G^{\prime }$ and $G^{\prime \prime }$ represent the elastic and viscous contributions of material. The bigger each moduli magnitude, the material behaves like an elastic solid or a viscous liquid (Barbucci et al., 2000; Koetting et al., 2015). In both hydrogel samples, $\;G^{\prime }$ was bigger than $G^{\prime \prime }$ in all frequencies, which means the hydrogels behave more like an elastic solid. $\;G^{\prime }$ and $G^{\prime \prime }$ curves had no intersect indicating that hydrogels had no phase transition and had stable viscoelastic behavior. In comparison, $\eta^{*}$ decreased with the addition of MNPs, and both hydrogels’ complex viscosity had decreased in the short timescales.

### 3.3 *In Vitro* Releasing Profile

**FIGURE 4:**
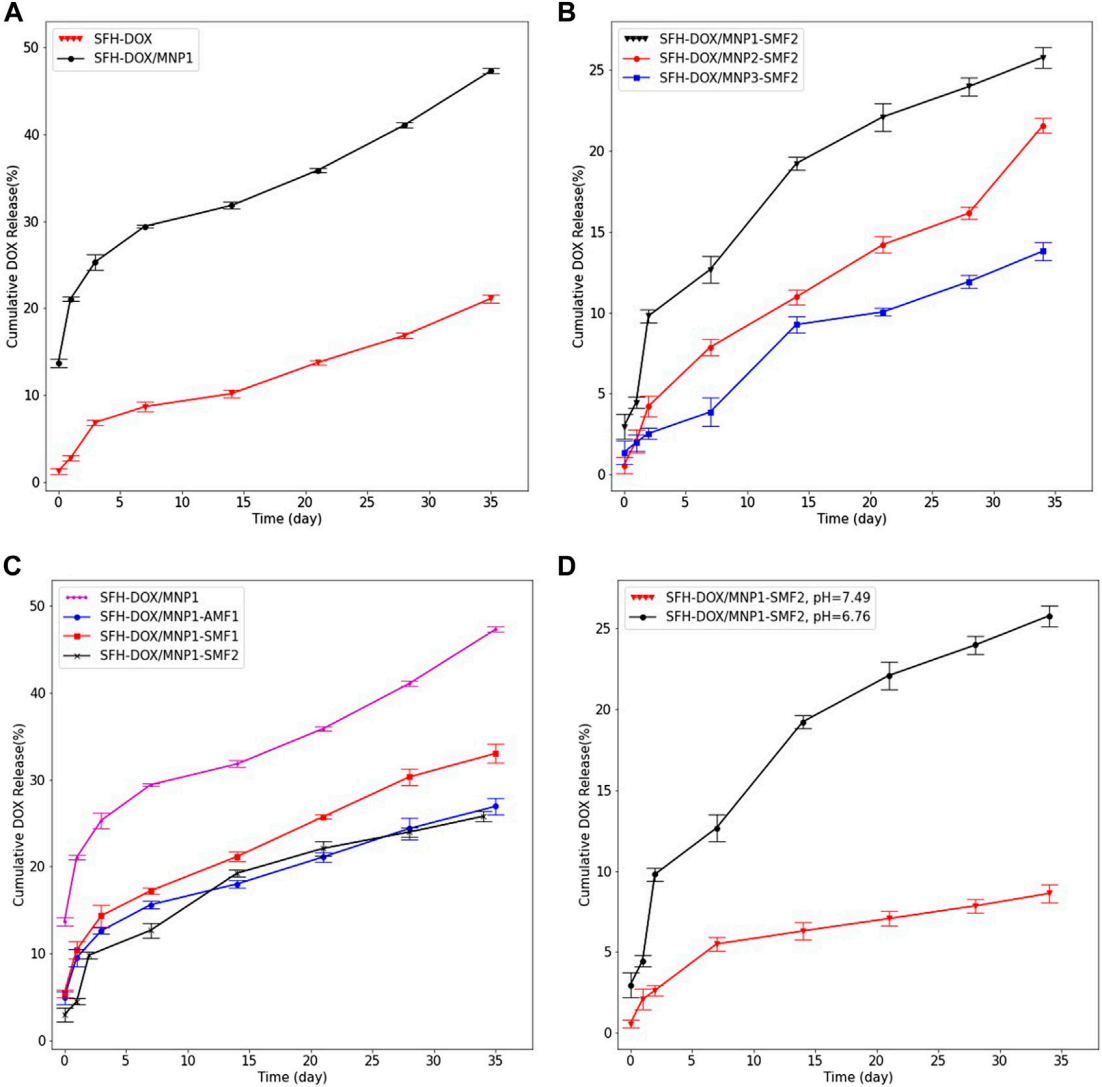
Evaluation of multiple stimuli-responsive properties of SFH. *In vitro* cumulative release profile of DOX from **(A)**; SFH and SFH/MNP1 in the absence of an EMF, **(B)**; different magnetic hydrogels containing different concentrations of MNPs, in the presence of 0.28 T SMF, **(C)**; SFH/MNP1 under the various EMF, **(D)**; SFH/MNP1 in two different release buffers acidity. Data are expressed as mean ± SD.

*In vitro* drug release pattern was investigated in eight different samples to study the effect of adding MNPs, the concentration of MNPs, applying an EMF, and pH in drug release from the SFH. In all samples, DOX was continuously released from the hydrogels for more than 35 days, representing the sustainable behavior of the drug release. No initial burst release was observed, meaning that all of the drugs loaded successfully in the SF scaffolds (Figure 4).

The influence of the addition of MNPs on the release of encapsulated DOX was investigated by studying the release of the DOX from SFH-DOX/MNP1 and compared with that of SFH-DOX. The result showed that the encapsulated DOX was released for a prolonged period from SFH-DOX compared to SFH-DOX/MNP1 in the absence of an EMF (Figure 4A). At 35 days, the cumulative release of DOX from SFH-DOX/MNP1 was more than twice faster (47.33%) than that without MNPs (21.12%). The difference in release patterns is closely associated with the viscosity and porosity, as described previously. The lower is the viscosity, the more accessible is the movement of drugs (Li et al., 2001). Moreover, diffusion from the pores is the primary mechanism of drug release (Peppas, 2000; Holback et al., 2011), and the addition of MNPs resulted in more porosity, thus, the drug release rate increased.

To study the effect of MNPs concentration on the release profile of DOX, three samples of SFH-DOX/MNP1, SFH-DOX/MNP2, and SFH-DOX/MNP3 were incubated in PBS 10X (pH 6.76), and SMF2 was applied for more than a month. Figure 4B shows that the cumulative release rate decreased with increasing MNPs in the formulation. We hypothesized that magnetic driven force caused direction movement of coated MNPs; thus, hydrogel pores were occupied because of the structure deformation, which reduced the drug release rate.

The SFH-DOX/MNP1 in the absence of EMF was taken as the control and compared with those under the SMF1, SMF2, and AMF1, to investigate the DOX cumulative release in the passive and EMF-induced mode (Figure 4C). The cumulative drug release was much higher in the absence of EMF than in the others. The *in vitro* release of DOX from SFH-DOX/MNP1 decreased with increasing the SMF strength. However, DOX release was more prevented in the presence of AMF1 compared with SMF1. This may contribute to coated magnetic nanoparticles’ orientation direction while applying a magnetic field. Pores are more likely to be blocked with the alternating direction of MNPs orientation than vertical orientation.

As presented in Figure 4D, after 34 days of incubation of SFH-DOX/MNP1 in the releasing media in the presence of SMF2, 8.62% of DOX was released in buffer (pH 7.49, mimicking blood plasma). However, much more DOX molecules (25.8%) were released in buffer (pH 6.76, extracellular environment in tumor site) compared to that in buffer with higher pH. These results demonstrated the inherent pH-responsibility of SFH-DOX (Gou et al., 2020), as investigated in previous studies. The enhancement in DOX release is due to weak electrostatic interactions between the drug and SF, in environments with lower pH (Duncan, 2003; Seib and Kaplan, 2012; Tian et al., 2014). Moreover, external factors like pH and temperature can affect drug release and polymer network degradation (de Moraes et al., 2015; Xu Z. et al., 2019; Gou et al., 2020). Therefore, the increase in drug release within lowering pH made this drug delivery system suitable for diminishing the side effects of chemotherapy by decreasing the drug accumulation in unfavorable sites.

### 3.4 Quantification of Cellular Viability

**FIGURE 5:**
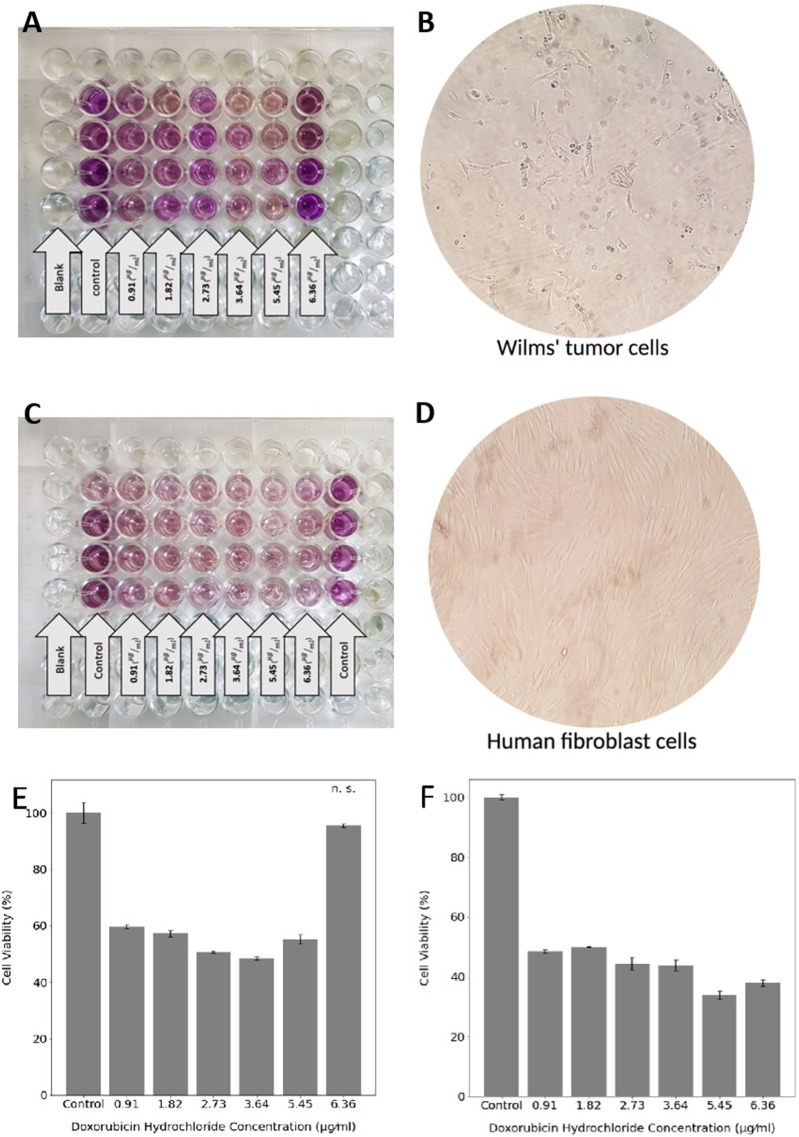
MTT assay of Wilms’ tumor and **(A)** and human fibroblast cells **(C)**. The morphology of Wilms’ tumor **(B)** and human fibroblast **(D)** cells under the microscope. Cell viability of Wilms’ tumor **(E)** and human fibroblast **(F)** in the exposure of six different DOX concentrations. Statistical differences between the control and other groups were determined with ANOVA, mean ± SD; *n* = 4, *p* < 0.05, n. s. = not significant.

For evaluating cell viability and proliferation in the exposure of different DOX concentrations released from different hydrogel samples, Wilms’ tumor (Figures 5A,B) and human fibroblast (Figures 5C,D) cells were incubated for 48 h. The resulting absorbance was proportional to the number of viable cells at each concentration; thus, the viability could be assessed. The metabolic activities of human fibroblast cells gradually decreased over the increasing DOX concentration (Figure 5F). However, Wilms’ tumor cells viability almost did not change (not statistically significant) in the exposure of the highest concentration of 6.36 μg/ml (Figure 5E). These results suggest that Wilms’ tumor cancer cells should be exposed to a controlled DOX concentration to prevent cell resistance. The IC50 values of DOX for Wilms’ tumor were calculated at 2.73 ± 0.004 (μg/ml), and at 1.82 ± 0.001 (μg/ml) for human fibroblast cells (Table 2). The higher IC50 value of DOX represents that a higher dose is needed for inhibiting Wilms’ tumor cells, while a lower dose of DOX can cause more death in normal cells, indicating the importance of a localized drug delivery approach for ameliorating the chemotherapy side effects.

**TABLE 2:** IC50 values for Wilms’ tumor and human fibroblast cells, treated with different DOX concentrations.

IC50 value	Wilms’ tumor cells	Human fibroblast cells
DOX (μg/ml)	2.73 ± 0.004	1.82 ± 0.001

IC50 values were determined by MTT assay (48 h of cultivation). Data were expressed as mean ± SD of four independent experiments, *p* < 0.05 compared to the control group.

## 4 Conclusion

The mechanical and rheological properties of SF hydrogel are reserved mainly with the addition of MNPs. The nanoparticle-assisted SF hydrogel introduces a 3D porous structure drug carrier with an extra function of magneto-responsive in addition to the pH and shear-responsive properties. These properties can be combined with governing the endogenous properties of SF, which significantly enable optimization of the drug release, providing more efficient therapy and minimizing the unwanted side effects. SF ferrogel with magneto-responsive ability has been prepared by introducing Fe3O4 nanoparticles to the SF solution through the blending method. This approach has developed as a drug delivery system with sustained and controlled release; this was achieved through simply tuning the concentration of MNPs and strength of the EMF. The *in vitro* drug release profile and MTT assay demonstrated that magnetic SF hydrogel works well as a novel drug delivery system in cancer therapy. This study proposes that controlled and sustained drug release can be achieved using optimization and regulation of magnetic SF hydrogels. Since SF hydrogels degrade faster in the conditions mimicking *in vivo* (Wang et al., 2008), such as the presence of enzymes and mechanical forces, this *in vitro* study was based on minimizing the drug release. Release assessment results pointed out that if magnetic SF hydrogel is used, an EMF should be applied; otherwise, it causes a higher drug release rate, which possibly leads to drug resistance. If lower drug release is favorable, the concentration of MNPs and the strength of the EMF should be maximized. Moreover, a low-frequency AMF with lower strength can reduce the release rate more in comparison to SMF. Future studies will focus on evaluating the drug release in mediums more mimicking the human body, in which the applied EMF is measurable *in vitro*. Numerical simulations also should be done for forecasting the drug release behavior, and the optimized magnetic hydrogels should be tested *in vivo*. Moreover, DOX can be combined with other chemotherapy drugs (both hydrophobic and hydrophilic) for Wilms’ tumor treatment, encapsulated in the hydrogels, and injected into the tumor site *in vivo*.

## Data Availability

The original contributions presented in the study are included in the article/Supplementary Material; further inquiries can be directed to the corresponding authors.
